# Acid Sphingomyelinase Gene Knockout Ameliorates Hyperhomocysteinemic Glomerular Injury in Mice Lacking Cystathionine-β-Synthase

**DOI:** 10.1371/journal.pone.0045020

**Published:** 2012-09-14

**Authors:** Krishna M. Boini, Min Xia, Justine M. Abais, Ming Xu, Cai-xia Li, Pin-Lan Li

**Affiliations:** Department of Pharmacology and Toxicology, Medical College of Virginia Campus, Virginia Commonwealth University, Richmond, Virginia, United States of America; Institut National de la Santé et de la Recherche Médicale, France

## Abstract

Acid sphingomyelinase (ASM) has been implicated in the development of hyperhomocysteinemia (hHcys)-induced glomerular oxidative stress and injury. However, it remains unknown whether genetically engineering of ASM gene produces beneficial or detrimental action on hHcys-induced glomerular injury. The present study generated and characterized the mice lacking cystathionine β-synthase (Cbs) and Asm mouse gene by cross breeding Cbs^+/−^ and Asm^+/−^ mice. Given that the homozygotes of Cbs^−/−/^Asm^−/−^ mice could not survive for 3 weeks. Cbs^+/−/^Asm^+/+^, Cbs^+/−/^Asm^+/−^ and Cbs^+/−/^Asm^−/−^ as well as their Cbs wild type littermates were used to study the role of Asm^−/−^ under a background of Cbs^+/−^ with hHcys. HPLC analysis revealed that plasma Hcys level was significantly elevated in Cbs heterozygous (Cbs^+/−^) mice with different copies of Asm gene compared to Cbs*^+/+^* mice with different Asm gene copies. Cbs^+/−/^Asm^+/+^ mice had significantly increased renal Asm activity, ceramide production and O_2_.^−^ level compared to Cbs^+/+^/Asm^+/+^, while Cbs^+/−/^Asm^−/−^ mice showed significantly reduced renal Asm activity, ceramide production and O_2_.^−^ level due to increased plasma Hcys levels. Confocal microscopy demonstrated that colocalization of podocin with ceramide was much lower in Cbs^+/−/^Asm^−/−^ mice compared to Cbs^+/−/^Asm^+/+^ mice, which was accompanied by a reduced glomerular damage index, albuminuria and proteinuria in Cbs^+/−/^Asm^−/−^ mice. Immunofluorescent analyses of the podocin, nephrin and desmin expression also illustrated less podocyte damages in the glomeruli from Cbs^+/−/^Asm^−/−^ mice compared to Cbs^+/−/^Asm^+/+^ mice. In *in vitro* studies of podocytes, hHcys-enhanced O_2_.^−^ production, desmin expression, and ceramide production as well as decreases in VEGF level and podocin expression in podocytes were substantially attenuated by prior treatment with amitriptyline, an Asm inhibitor. In conclusion, Asm gene knockout or corresponding enzyme inhibition protects the podocytes and glomeruli from hHcys-induced oxidative stress and injury.

## Introduction

Acid sphingomyelinase (ASM), a ceramide producing enzyme has been reported to be involved in the regulation of cell and organ functions and has been implicated in the development of different diseases such as obesity, diabetes, atherosclerosis, kidney diseases and disorder of lipid metabolism [1]–[3]. ASM hydrolyzes sphingomyelin to ceramide and phosphorylcholine and thereby exerts its signaling or regulatory role. It has been reported that ASM deficiency leads to Niemann-Pick disease in humans and that Asm gene (Asm is commonly used to represent mouse gene for ASM) knockout in mice resulted in the resistance to radiation [4] and other forms of stress-induced apoptosis [1]. Similarly, inhibition of ASM activity has also been shown to render cells and animals resistant to the apoptotic effects of diverse stimuli including Fas/CD95 [5], ischemia [6], radiation [7], chemotherapy [8] tumor necrosis factor-alpha (TNF-α) [9]. In addition, Asm knockout or Asm inhibition was shown to have protective action during the lung inflammation and fibrosis [10], cystic fibrosis [11]–[12], obesity and associated glomerular injury [13], liver fibrogenesis [14] and renal fibrosis [15].

In recent studies, we and others have demonstrated that ASM can be activated during hHcys whereby ceramide is produced to result in activation of NADPH oxidase, local oxidative stress and consequent glomerulosclerosis and loss of kidney functions [16]–[19]. However, most of these studies were done using pharmacological or molecular interventions, but to our knowledge no genetic approaches have been used to address the role of ASM-ceramide regulatory mechanism in the development of hHcys-associated glomerular injury or end-stage renal disease. Recently, the characterization of Cbs gene knockout mice as one of the hHcys model and development of Asm gene deletion in mice [20]–[21] provide opportunity to address whether genetically manipulation of both genes can alter hHcys-induced pathological changes, in particular in the renal glomeruli, which is a major focus of our laboratory. In the present study, we hypothesized that genetically engineering of Asm gene protects glomeruli from hHcys-induced glomerular oxidative stress and thereby ameliorate podocyte injury and glomerulosclerosis during hHcys. To test this hypothesis, we for the first time generated the mice lacking Asm and Cbs gene (lacking one alle of Cbs and two alle of Asm genes) to determine whether Asm deletion has any affect on glomerular oxidative stress and podocyte injury by hHcys that is occurred in Cbs gene deficient mice. By analysis of Asm homozygous and heterozygous mice with a background of Cbs partially deletion, we tried to obtain gene titration data clarifying the pathogenic role in hHcys. Using culture murine podocytes, we further examined the direct effects of ASM inhibition on Hcys-induced cellular oxidative stress and related injury. These *in vivo* and *in vitro* experiments elucidate the role of ASM in the development of podocyte injury and glomerular sclerosis associated with hHcys, which may identify an important target for possible gene therapy during the course of hHcys-induced pathology.

## Results

### Genotyping and Plasma Hcys Concentrations in DKO Mice

**Figure 1:**
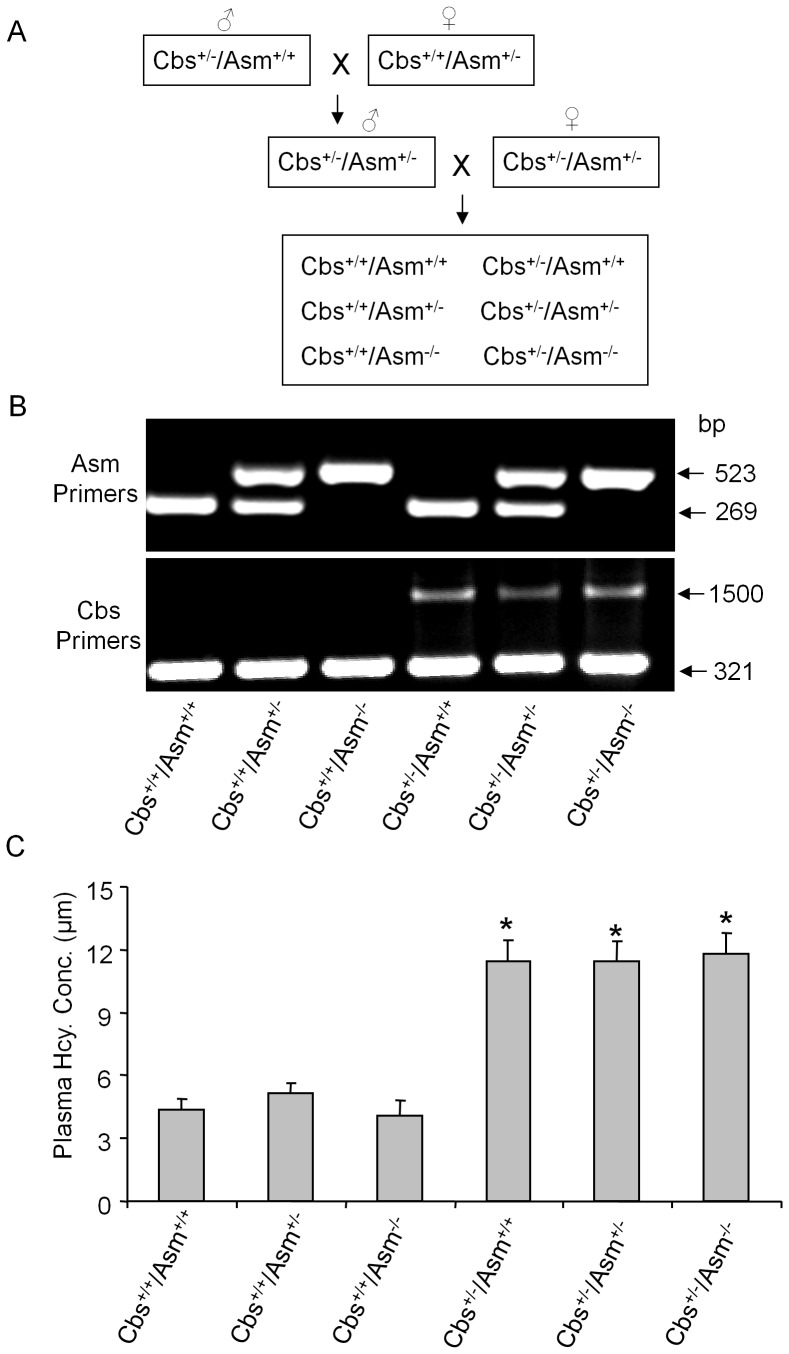
Genotyping and Plasma Hcys concentrations in Cbs^+/+^/Asm^+/+^, Cbs^+/+^/Asm^+/−^, Cbs^+/+^/Asm^−/−^, Cbs^+/−/^Asm^+/+^, Cbs ^+/−/^Asm^+/−^ and Cbs^+/−/^Asm^−/−^ mice. A: Breeding scheme used to generate Cbs^+/+^/Asm^+/+^, Cbs^+/+^/Asm^+/−^, Cbs^+/+^/Asm^−/−^, Cbs^+/−/^Asm^+/+^, Cbs^+/−/^Asm^+/−^ and Cbs^+/−/^Asm^−/−^ mice. B: Genotyping in mutant mice. Two PCR products suggest that heterozygous mutation, while single band represents wild type or knockout allele. C: Values are arithmetic means ± SE (n = 6 each group) of plasma Hcys concentrations in Cbs^+/+^/Asm^+/+^, Cbs^+/+^/Asm^+/−^, Cbs^+/+^/Asm^−/−^, Cbs^+/−/^Asm^+/+^, Cbs^+/−/^Asm^+/−^ and Cbs^+/−/^Asm^−/−^ mice. * Significant difference (*P*<0.05) compared to the values from Cbs^+/+^/Asm^+/+^ mice; ^#^ Significant difference (*P*<0.05) compared to the values from Cbs^+/−/^Asm^+/+^ mice.

The genotypes of the mutant mice were confirmed by PCR using Cbs and Asm mouse gene specific primers. As shown in Figure 1B, when Cbs gene primer was used for genotyping, a 321 bp and 1500 bp product could be detected. Mice with only a 321 bp band are wild type (Cbs^+/+^), while mice with two bands are heterozygotes (Cbs^+/−^). In Asm genotyping, mice showing a single product of 269 or 523 bp indicate Asm wild type (Asm^+/+^) and homozygotes (Asm^−/−^), respectively. If both the two products was detected in the same mice, these mice were heterozygotes of Asm gene (Asm^+/−^). HPLC analyses showed that the plasma Hcys concentrations was similar among Cbs^+/+^/Asm^+/+^, Cbs^+/+^/Asm^+/−^ and Cbs^+/+^/Asm^−/−^ mice, which showed different Asm gene types with the same background of Cbs wild type. Compared to these Cbs wild type mice, the plasma Hcys concentrations were significantly increased in Cbs heterozygotes with different Asm gene copies, namely, Cbs^+/−/^Asm^+/+^, Cbs^+/−/^Asm^+/−^ and Cbs^+/−/^Asm^−/−^ mice, but there was no significant difference in plasma Hcys levels in this group of mice with Cbs^+/−^ background with different copies of Asm gene (Figure 1C). These data suggest that Asm gene is not involved in the regulation of plasma Hcys levels and therefore it does not alter the occurrence of hHcys in mice.

**Figure 2:**
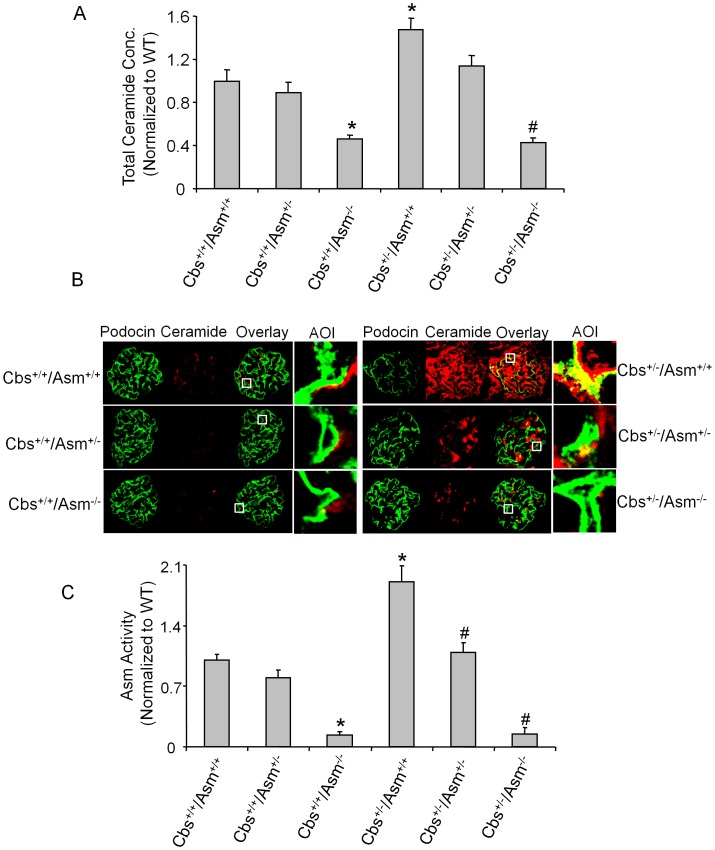
Renal tissue ceramide production and Asm activity in Cbs^+/+^/Asm^+/+^, Cbs^+/+^/Asm^+/−^, Cbs^+/+^/Asm^−/−^, Cbs^+/−/^Asm^+/+^, Cbs^+/−/^Asm^+/−^ and Cbs^+/−/^Asm^−/−^ mice. Values are arithmetic means ± SE (n = 6 each group) of total ceramide concentrations (A), ceramide production (B) and Asm activity (C) in Cbs^+/+^/Asm^+/+^, Cbs^+/+^/Asm^+/−^, Cbs^+/+^/Asm^−/−^, Cbs^+/−/^Asm^+/+^, Cbs^+/−/^Asm^+/−^ and Cbs^+/−/^Asm^−/−^ mice. The AOI shows the higher magnification of boxed area in overlaid images in panel B. AOI: Area of interest. * Significant difference (*P*<0.05) compared to the values from Cbs^+/+^/Asm^+/+^ mice; ^#^ Significant difference (*P*<0.05) compared to the values from Cbs^+/−/^Asm^+/+^ mice.

As illustrated in Figure 2A, the total renal ceramide levels were substantially lower in Cbs^+/+^/Asm^−/−^ than Cbs^+/+^/Asm^+/+^ and Cbs^+/+^/Asm^+/−^ mice. However, increased plasma Hcys level was accompanied by significantly increased renal ceramide levels in Cbs^+/−/^Asm^+/+^ mice, but not in Cbs^+/−/^Asm^−/−^ mice. Furthermore, confocal microscopic studies revealed that ceramide staining was more pronounced in glomeruli of Cbs^+/−/^Asm^+/+^ mice than in Cbs^+/−/^Asm^−/−^ mice (Figure 2B). In addition, using podocin as a podocyte marker we showed that hHcys-induced ceramide expression in glomeruli were mostly located in podocytes, as demonstrated by the colocalization of ceramide with podocin. This colocalization was substantially blocked in Cbs^+/−/^Asm^−/−^ mice (Figure 2B). The Asm activity was significantly lower in Cbs^+/+^/Asm^−/−^ compared to Cbs^+/+^/Asm^+/+^ mice, but it was significantly increased in Cbs^+/−/^Asm^+/+^ compared to Cbs^+/+^/Asm^+/+^ mice. However, this hHcys-induced Asm activity was significantly attenuated in Cbs^+/−/^Asm^−/−^ mice (Figure 2C).

### Attenuation of hHcys-induced Glomerular Injury in Cbs^+/−^ by Further Knockout of Asm Gene

**Figure 3:**
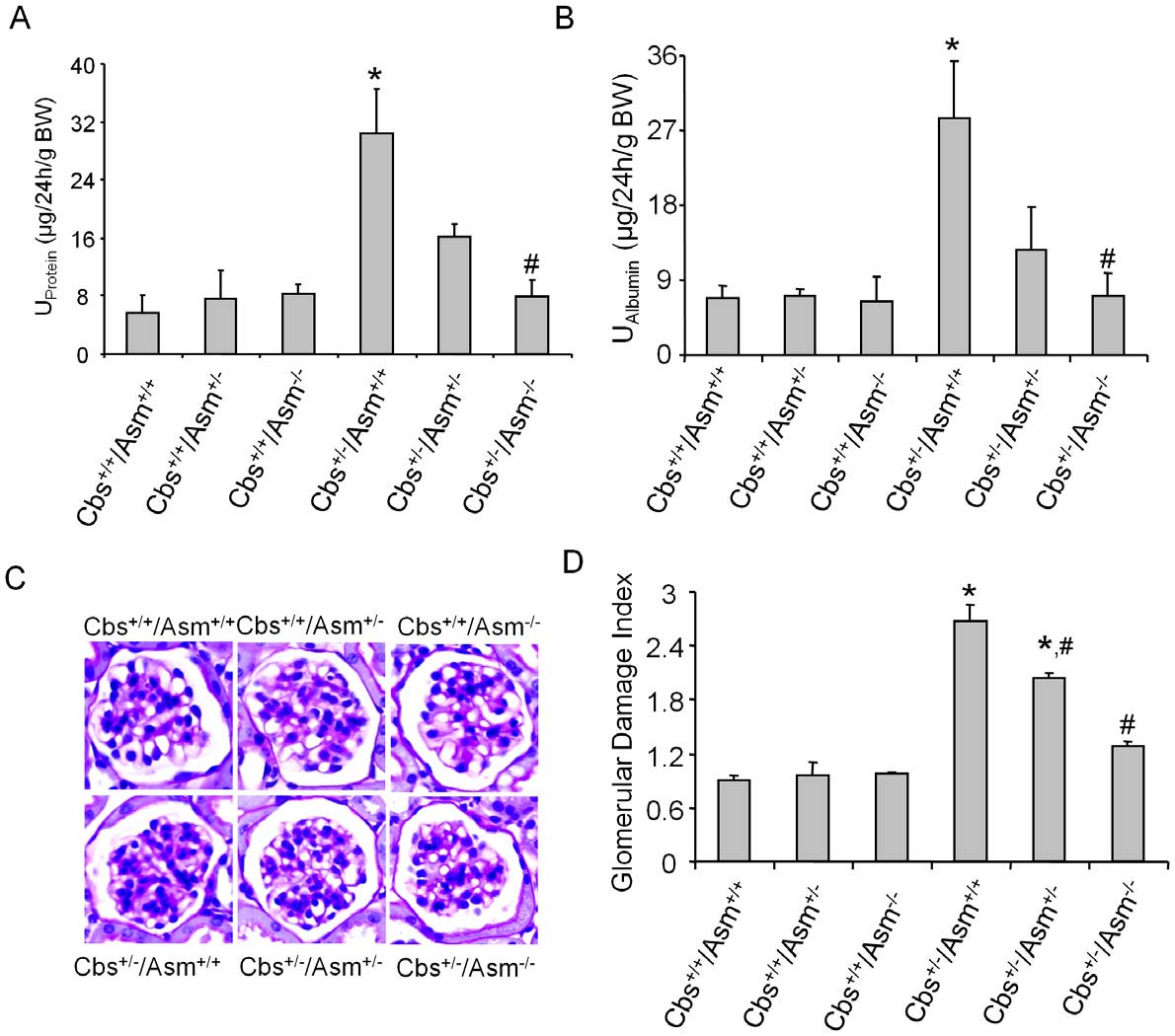
Glomerular injury in Cbs^+/+^/Asm^+/+^, Cbs^+/+^/Asm^+/−^, Cbs^+/+^/Asm^−/−^, Cbs^+/−/^Asm^+/+^, Cbs^+/−/^Asm^+/−^ and Cbs^+/−/^Asm^−/−^ mice. A: Values are arithmetic means ± SEM (n = 6 each group) of urinary total protein excretion, B: Urinary albumin excretion in Cbs^+/+^/Asm^+/+^, Cbs^+/+^/Asm^+/−^, Cbs^+/+^/Asm^−/−^, Cbs^+/−/^Asm^+/+^, Cbs^+/−/^Asm^+/−^ and Cbs^+/−/^Asm^−/−^ mice. C: Photomicrographs show typical glomerular structure (original magnification, x400) in Cbs^+/+^/Asm^+/+^, Cbs^+/+^/Asm^+/−^, Cbs^+/+^/Asm^−/−^, Cbs^+/−/^Asm^+/+^, Cbs^+/−/^Asm^+/−^ and Cbs^+/−/^Asm^−/−^ mice. D: Summarized data of glomerular damage index (GDI) by semi-quantitation of scores in 6 different groups of mice (n = 6 each group). For each kidney section, 50 glomeruli were randomly chosen for the calculation of GDI. * Significant difference (*P*<0.05) compared to the values from Cbs^+/+^/Asm^+/+^ mice; ^#^ Significant difference (*P*<0.05) compared to the values from Cbs^+/−/^Asm^+/+^ mice.

As depicted in Figure 3A and 3B, urinary protein and albumin excretion was similar in Cbs^+/+^/Asm^+/+^, Cbs^+/+^/Asm^+/−^ and Cbs^+/+^/Asm^−/−^ mice. However, the urinary total protein and albumin excretion increased significantly in Cbs^+/−/^Asm^+/+^ mice, but not in Cbs^+/−/^Asm^−/−^ mice, despite that both mouse strains have hHcys. By PAS staining, we observed a typical pathological change showing glomerular sclerotic damage in Cbs^+/−/^Asm^+/+^ mice such as glomerular capillary collapse and mesangial expansion. This pathology was not observed in Cbs^+/−/^Asm^−/−^ mice. The average glomerular damage index (GDI) was significantly higher in Cbs^+/−/^Asm^+/+^ mice than in Cbs^+/−/^Asm^−/−^ mice (Figure 3C and 3D).

**Figure 4:**
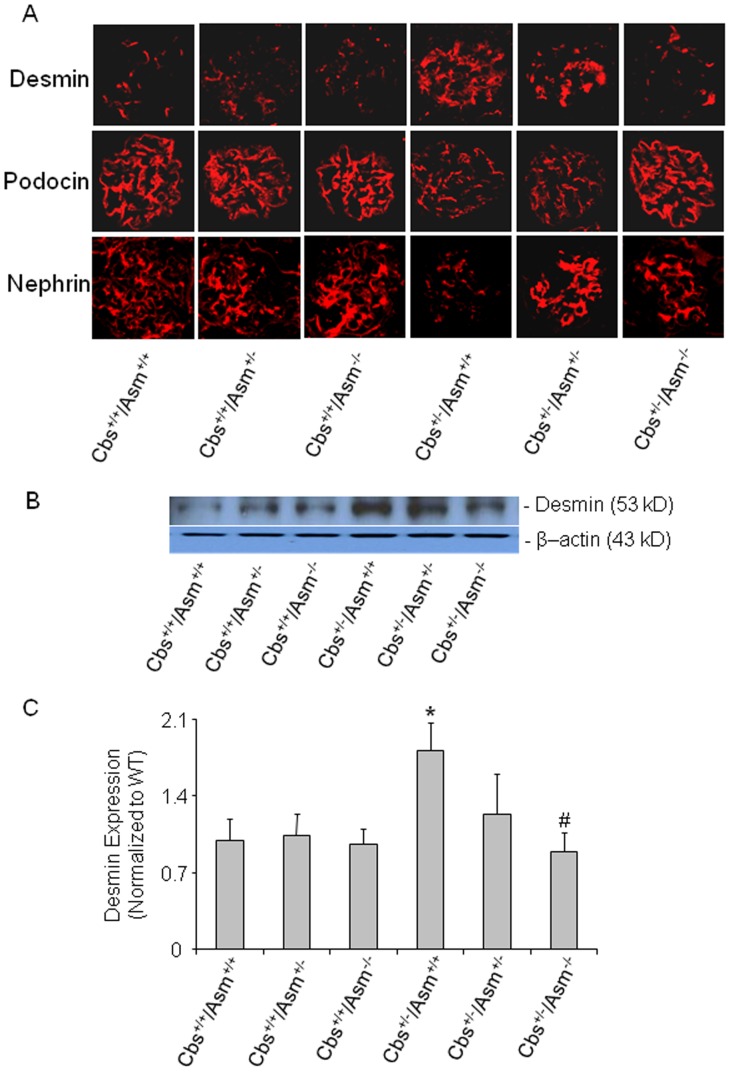
Immunofluorescent staining of anti-desmin, anti-podocin and anti-nephrin antibody in glomeruli of Cbs^+/+^/Asm^+/+^, Cbs^+/+^/Asm^+/−^, Cbs^+/+^/Asm^−/−^, Cbs^+/−/^Asm^+/+^, Cbs^+/−/^Asm^+/−^ and Cbs^+/−/^Asm^−/−^ mice. A: Typical images of desmin, podocin and nephrin staining in glomeruli from Cbs^+/+^/Asm^+/+^, Cbs^+/+^/Asm^+/−^, Cbs^+/+^/Asm^−/−^, Cbs^+/−/^Asm^+/+^, Cbs^+/−/^Asm^+/−^ and Cbs^+/−/^Asm^−/−^ mice (n = 6 each group). B: Western blot analysis of desmin protein expression in different groups of mice. C: Summarized data showing the quantification of desmin expression, which was normalized to β-actin (n = 6). * Significant difference (*P*<0.05) compared to the values from Cbs^+/+^/Asm^+/+^ mice; ^#^ Significant difference (*P*<0.05) compared to the values from Cbs^+/−/^Asm^+/+^ mice.

Further, we determined whether protective effect of Cbs^+/−/^Asm^−/−^ gene knockout is associated with the protection of podocytes in hHcys-induced glomerular injury. As shown in Figure 4A, the immunofluorescent analysis showed that desmin staining was more pronounced in glomeruli of Cbs^+/−/^Asm^+/+^ than Cbs^+/+^/Asm^+/+^ and Cbs^+/+^/Asm^+/−^ mice, as shown by more remarkable red fluorescence detected in glomeruli, which indicates podocytes injury. However in Cbs^+/−/^Asm^−/−^ mice the hHcys-induced increase in desmin expression within glomeruli of mice was not observed (Figure 4A). In contrast, other podocyte markers podocin and nephrin staining were found markedly reduced in glomeruli of Cbs^+/−/^Asm^+/+^ mice compared to Cbs^+/+^/Asm^+/+^ and Cbs^+/+^/Asm^+/−^ mice, and in Cbs^+/−/^Asm^−/−^ mice this reduced podocin and nephrin expression or production were not observed (Figure 4A). In addition, Western blot analysis demonstrated that desmin protein expression was significantly increased in Cbs^+/−/^Asm^+/+^ mice compared to Cbs^+/+^/Asm^+/+^. However, the enhanced desmin expression was attenuated in Cbs^+/−/^Asm^−/−^ mice (Figure 4B and 4C).

### Lack of Local Oxidative Stress in the Glomeruli of Cbs^+/−/^Asm^−/−^ Mice with hHcys

**Figure 5:**
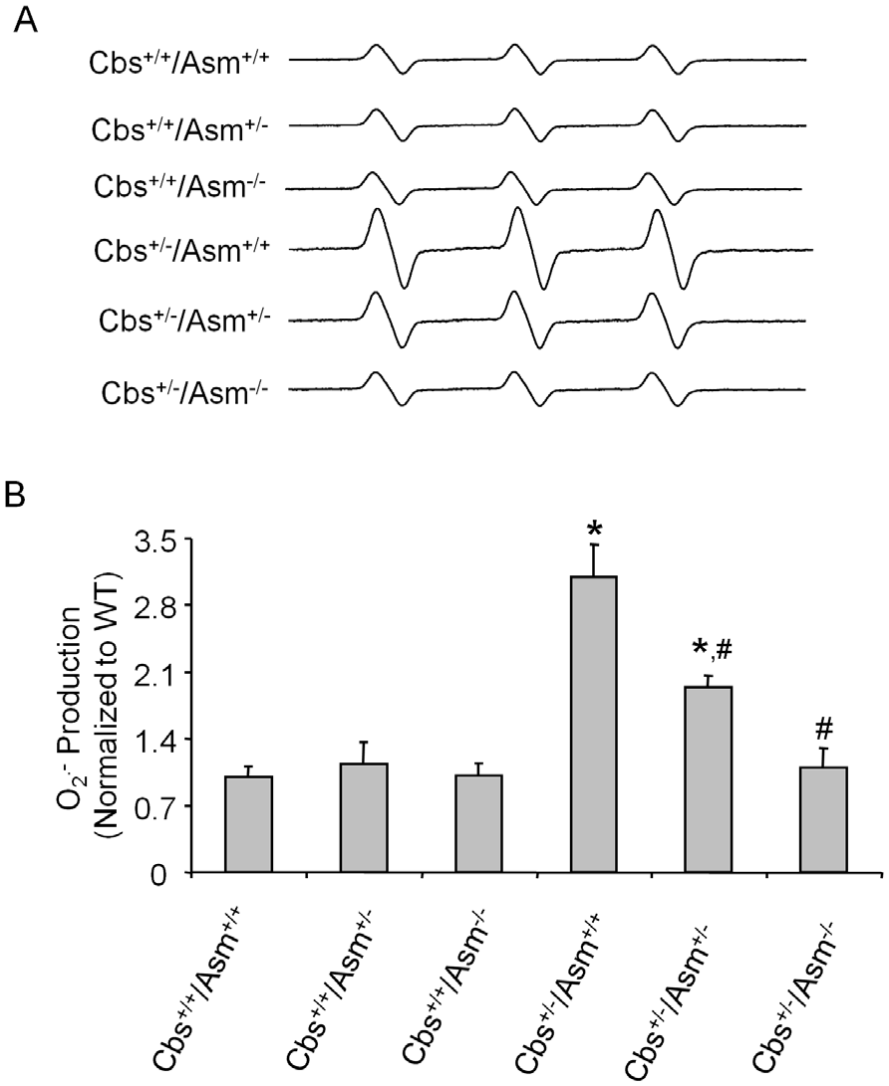
Glomerular O_2_.^−^ production in Cbs^+/+^/Asm^+/+^, Cbs^+/+^/Asm^+/−^, Cbs^+/+^/Asm^−/−^, Cbs^+/−/^Asm^+/+^, Cbs^+/−/^Asm^+/−^ and Cbs^+/−/^Asm^−/−^ mice. A: Representative ESR spectra traces for O_2_.^−^ production in 6 different groups of mice. B: Values are arithmetic means ± SEM (n = 5 each group) of O_2_.^−^ production in Cbs^+/+^/Asm^+/+^, Cbs^+/+^/Asm^+/−^, Cbs^+/+^/Asm^−/−^, Cbs^+/−/^Asm^+/+^, Cbs^+/−/^Asm^+/−^ and Cbs^+/−/^Asm^−/−^. * Significant difference (*P*<0.05) compared to the values from Cbs^+/+^/Asm^+/+^ mice; ^#^ Significant difference (*P*<0.05) compared to the values from Cbs^+/−/^Asm^+/+^ mice.

As illustrated in Figure 5A, the ESR spectrometric curve exhibited significant increase in the amplitude of Nox-dependent ESR O_2_.^−^ signals in the glomeruli of Cbs^+/−/^Asm^+/+^ mice compared to mice with Cbs^+/+^ background and different copies of Asm genes. However, this enhanced glomerular O_2_.^−^ production in Cbs^+/−/^Asm^+/+^ mice was not observed in Cbs^+/−/^Asm^−/−^ mice. These results were summarized in Figure 5B, showing that glomerular O_2_.^−^ production was similar in all Cbs^+/+^ mice with different Asm gene copies, but it was increased by 3.1 folds in Cbs^+/−/^Asm^+/+^ mice compared to those mice with Cbs^+/+^ and different Asm gene copies. This hHcys-induced glomerular O_2_.^−^ level enhanced in Cbs^+/−/^Asm^+/+^ mice were attenuated in Cbs^+/−/^Asm^−/−^ mice.

### Blockade of Hcys-induced Ceramide Expression and Podocyte Injury by Asm Inhibition in Cultured Podocytes

**Figure 6:**
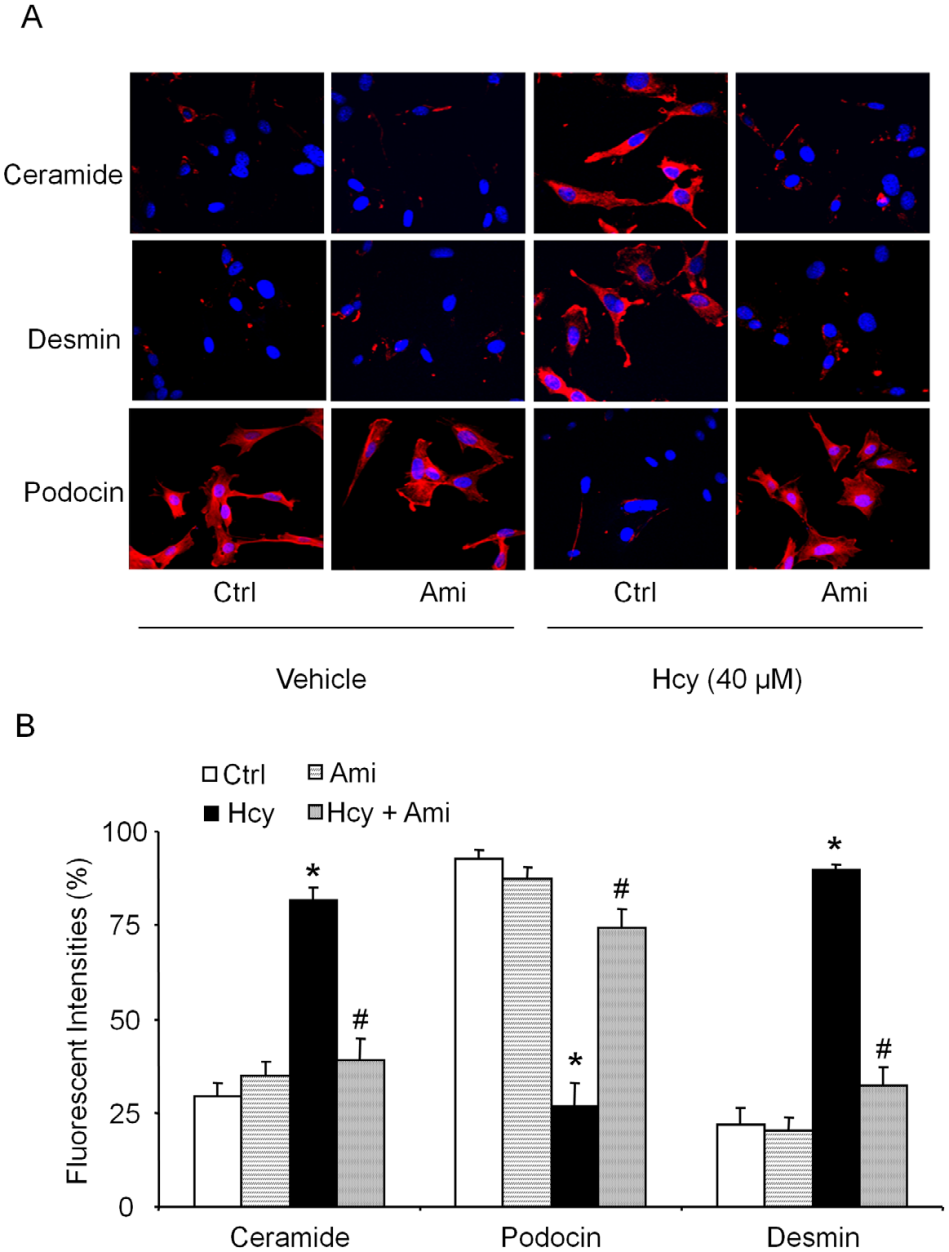
Production of ceramide, podocin and desmin staining in podocytes with or without Hcys and/or amitriptyline treatment. Cultured podocytes were treated with or without Hcys for 24 h. A: Typical fluorescent microscopic images of ceramide, podocin and desmin in Hcys and/or amitriptyline treated podocytes (n = 5/group, Original magnification, ×400). B: Values are arithmetic means ± SE (n = 5 each group) of summarized data showing the percentage of positive staining cells of ceramide, desmin and podocin *vs*. control group. Ctrl: Control, Hcy: Homocysteine, Ami: Amitriptyline. * Significant difference (*P*<0.05) compared to the control group; ^#^ Significant difference (*P*<0.05) compared to the Hcys group.

The above studies demonstrated that mice lacking Cbs and Asm gene protect glomerular oxidative stress, injury and podocyte injury. We further performed some *in vitro* experiments to confirm whether glomerular injury truly occurs in podocytes. Using cultured murine podocytes, we examined ceramide production and related expression of podocyte markers. As shown in Figure 6, immunofluorescent analysis demonstrated that Hcys stimulation increased the desmin and ceramide expression in podocytes compared to untreated cells. Prior treatment with Asm inhibitor, amitriptyline decreased Hcys-induced elevation of desmin and ceramide production in podocytes (Figure 6A). Another podocyte marker, however, podocin was markedly reduced upon Hcys stimulation in podocytes, and the Asm inhibition almost completely attenuated the decrease in podocin expression (Figure 6A). The summarized data were shown in Figure 6B.

### Blockade of Hcys-induced Oxidative Stress and Decrease in VEGF-A Secretion by Asm Inhibition

**Figure 7:**
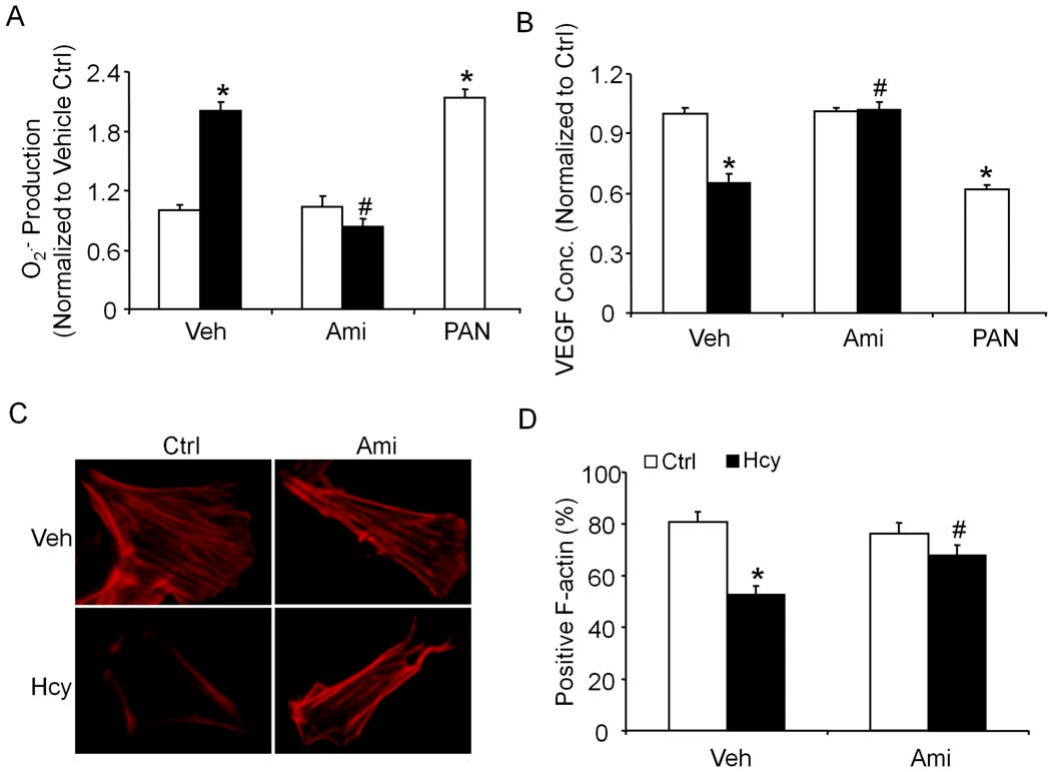
O_2_.^ –^ production and VEGF levels in podocytes with or without Hcys and/or amitriptyline treatment. Cultured podocytes were treated with or without Hcys for 24 h. Values are arithmetic means ± SE (n = 5 each group) of O_2_.^−^ production (A), VEGF levels (B) in podocytes with or without Hcys and/or amitriptyline treatment. C. Representative microscopic images showing F-actin staining in podocytes using rhodamine-phalloidin staining (magnification, ×400). D. Summarized data showing the rate of podocytes retaining distinct longitudinal stress fibers. Scoring was from 100 podocytes on each slide in different groups (n = 5 each group). Ctrl: Control, Hcy: Homocysteine, Ami: Amitriptyline, Veh: Vehicle. * Significant difference (*P*<0.05) compared to the control group; ^#^ Significant difference (*P*<0.05) compared to the Hcys group.

As depicted in Figure 7A, the O_2_.^−^ production was similar in both control and amitriptyline-treated podocytes. However, the Hcys stimulation significantly increased the O_2_.^−^ production by 1.9 folds compared to control cells, but prior treatment with amitriptyline significantly attenuated the Hcys-induced O_2_.^−^ production (Figure 7A). As a functional parameter of podocytes, VEGF-A production was detected in podocytes under various conditions. It was found that VEGF-A production and secretion were dramatically decreased by L-Hcys treatment compared to control cells. However, pretreatment of podocytes with amitriptyline significantly attenuated the Hcys-induced VEGF-A secretion. PAN is served a positive control (Figure 7B). Furthermore, we also performed the immunofluorescent experiments using rhodamine-phalloidin as a dye for F-actin, it was found that Hcys treatment induced dramatic reorganization and disruption of the F-actin cytoskeleton in podocytes compared to control cells. However, pretreatment of podocytes with amitriptyline attenuated the Hcys-induced disruption of F-actin cytoskeleton in podocytes and were restored to normal (Figure 7C and 7D).

## Discussion

The major goal of the present study is to determine whether genetically engineering of acid sphingomyelinase (Asm) produces beneficial or detrimental effects in the development of hHcys-induced glomerular injury and sclerosis. We found that the genetic model of hyperhomocysteinemic Cbs mice (Cbs^+/−/^Asm^+/+^) enhanced the ceramide production and Asm activity, which was attributed to NADPH oxidase dependent O_2_.^−^ production and local oxidative stress in glomeruli and ultimately led to podocyte injury and glomerulosclerosis. These results demonstrate for the first time that mice lacking Cbs and Asm gene (Cbs^+/−/^Asm^−/−^) protect against the hHcys-induced glomerular oxidative stress and injury in mice.

We first generated and characterized the mice lacking cystathionine β-synthase (Cbs) and acid sphingomyelinase (Asm) gene by cross breeding Cbs^+/−^ and Asm^+/−^ mice. Given that the homozygotes of Cbs^−/−/^Asm^−/−^ mice could not survive for 3 weeks, Cbs^+/−/^Asm^+/+^, Cbs^+/−/^Asm^+/−^ and Cbs^+/−/^Asm^−/−^ as well as their Cbs wild type littermates were used to study the role of Asm^−/−^ under a background of Cbs^+/−^ that produced hHcys. Previous studies have shown that Hcys levels in the blood are a complex trait that is affected by several genetic and environmental factors. It is known that genetic factors contribute to mild, moderate [22] and severe hHcys [23], and that the genetic background is the specific collection of allelic gene variants that make individuals to present different inheritable characters within species. In this sense, inbred mouse strains are widely used to study the effects of different genetic background on the disease phenotype [24]. In the present study, we tested the role of Asm gene in the development of hHcys-induced glomerular injury or sclerosis by using Cbs^+/−^ mutant mice. These mice have 50% reduction in Cbs mRNA and enzyme activity in the liver and have a normal plasma Hcys levels by 2 folds higher than that in wild type littermates [19], [25]. Thus, the Cbs^+/−^ mice may develop mild hHcys and are a good model to study the hHcys-related disease processes [19]. Indeed, our results showed that plasma Hcys concentration was two fold higher in all Cbs^+/−/^Asm^+/+^, Cbs^+/−/^Asm^+/−^ and Cbs^+/−/^Asm^−/−^ mice compared to their Cbs wild type littermates. These results suggest that Asm itself is not involved in the metabolism of Hcys. Importantly, we found that the increased plasma Hcys concentration resulted in a remarkable glomerular damage or sclerosis in Cbs^+/−/^Asm^+/+^ mice, but not in Cbs^+/−/^Asm^−/−^ mice, suggesting that Asm gene knocking out prevents glomeruli from hHcys-induced injury in mice with fewer copies of Cbs genes.

There is considerable evidence supporting the critical role of ceramide signaling pathway in the pathogenesis of kidney diseases [18], [21]. Ceramide production is mainly mediated via the hydrolysis of membrane sphingomyelin by various sphingomyelinases such as acid sphingomyelinase (Asm) or neutral sphingomyelinase (NSM) or by *de novo* synthesis via serine palmitoyltransferase (SPT) and ceramide synthase [26]. It is subsequently metabolized into sphingosine by ceramidases, and sphingosine can be further converted to S1P via sphingosine kinase [26] in response to a variety of stimuli including proinflammatory cytokines, oxidative stress, and increased levels of free fatty acids. It has been reported that ceramide may mediate the detrimental or pathogenic actions induced by many different injury factors in different cells and tissues [27]–[29]. More recently, ceramide-mediated signaling has been found to cross talk with redox signaling associated with NAD(P)H oxidase, which represents a novel cellular signaling cascade that participates in the development of different diseases [18], [21]. In this regard, we recently reported that increased plasma Hcys concentrations enhanced the ceramide production leading to activation of NAD(P)H oxidase in the kidney and that inhibition of ceramide production improved glomerular injury in those hyperhomocysteinemic rats [18]. The present study further demonstrated that Asm gene knockout attenuated hHcys-induced ceramide production, local oxidative stress and glomerular injury in mice lacking Cbs gene (Cbs^+/−/^Asm^−/−^).

Using podocin as a podocytes marker, our confocal microscopic data showed that hHcys-induced ceramide expression in glomeruli was mostly located in podocytes, as demonstrated by the colocalization of ceramide with podocin. This colocalization was substantially blocked in mice lacking both Asm and Cbs gene (Cbs^+/−/^Asm^−/−^). Furthermore, Asm activity in renal tissues were significantly increased in hyperhomocysteinemic Cbs^+/−/^Asm^+/+^ mice, but not in Cbs^+/−/^Asm^−/−^. The increased Asm activity in Cbs^+/−/^Asm^+/+^ mice may at least be partially due to the enhancement of Asm mRNA expression. In this regard, our previous studies have shown that hHcys increased the Asm activity and Asm mRNA expression in renal tissues of Asm^+/+^ mice but not in Asm^−/−^ mice [21]. It was also shown that Hcys stimulation in podocytes enhanced the co-localization of membrane raft and Asm in the plasma membrane and revealed the translocation of Asm into cell membrane upon Hcys treatment [21]. These results suggest that hHcys-induced renal and glomerular ceramide production is mainly caused by activation of Asm in mice. In accordance with lowered ceramide production in Cbs^+/−/^Asm^−/−^ mice, urinary albumin, protein excretion and glomerular injury and sclerosis were also significantly decreased compared with Cbs^+/−/^Asm^+/+^ mice, suggesting that ceramide-associated renal injury during hHcys is alleviated in these Cbs^+/−/^Asm^−/−^. Taken together, these results suggest that Asm gene knockout produces the beneficial effects in hyperhomocysteinemic mice that lack Cbs gene and therefore Asm gene and corresponding signaling pathway could be a therapeutic target for hHcys-induced podocyte injury and consequent glomerular sclerosis.

To further explore the mechanisms by which Asm gene knockout protects glomeruli from injury induced by hHcys, we observed more changes in podocyte function in various gene mutant mice. It has been well documented that proteinuria is a hallmark of renal injury and a major deteriorating factor for progression of end-stage renal diseases [30]. The outer aspect of glomerular basement membrane is lined up with very specialized visceral epithelial cells, named podocytes, and these podocytes serve as the final defense against urinary protein loss in the normal glomerulus [31]. Any damage to these podocytes and their slit diaphragm is intimately associated with proteinuria [32]. The assessment of normal slit diaphragm component such as podocin [33] and injured podocyte marker, desmin [34] are now therefore considered as two major sensitive markers of podocyte injury and subsequently glomerulopathy in renal diseases. In the present study, we showed that podocin and nephrin proteins were markedly decreased in hyperhomocysteinemic Cbs^+/−/^Asm^+/+^ mice, but not in mice lacking both Asm and Cbs gene (Cbs^+/−/^Asm^−/−^). In addition, we found that desmin was markedly increased in the glomeruli of Cbs^+/−/^Asm^+/+^ mice compared to Cbs^+/−/^Asm^−/−^ mice. These results further support the view that hHcys-induced glomerular injury is associated with increased ceramide production via Asm and its pathological action on podocytes. Furthermore, several studies have demonstrated that NADPH oxidase-dependent O_2_.^−^ production is an early event for Hcys-induced glomerular cell damage and glomerular sclerosis [18], [35]. It is possible that hHcys-induced NADPH oxidase activation is mediated by enhanced Asm activity in Cbs^+/−/^Asm^+/+^ mice. To test this hypothesis, the present study demonstrated by electron spin resonance analysis that hHcys indeed significantly increased NADPH oxidase-dependent O_2_.^−^ production in Cbs^+/−/^Asm^+/+^ mice, but not in Cbs^+/−/^Asm^−/−^ mice. These results support the view that Asm gene expression and ceramide production play a critical role in mediating glomerular O_2_.^−^ production through activation of NADPH oxidase during hHcys.

In addition to the whole animal experiments, we also used cultured murine podocytes to examine the direct effect of altered ASM activity on ceramide production and podocyte injury, which attempted to further confirm the role of ceramide and consequent NADPH oxidase activation in Hcys-induced podocyte injury. It was found that Hcys stimulation in mouse podocytes significantly increased the ceramide and desmin expression, but decreased the podocin expression compared to control cell group. However, pretreatment with amitriptyline, an Asm inhibitor attenuated the Hcys-induced ceramide production and podocyte injury. Furthermore, we examined whether the effects of ASM inhibition are associated with Hcys-enhanced oxidative stress in podocytes. It was found that amitriptyline blocked Hcys-induced NADPH oxidase activation. Given that ceramide production is a critical early mechanism initiating or promoting Hcys-induced podocyte injury and glomerulosclerosis [36], these results from cultured mouse podocytes further confirm the findings from our *in vivo* studies, supporting a conclusion that Hcys-induced podocyte and glomerular injury is associated with increased ceramide production via ASM activity. Another functional abnormality of Hcys-induced podocyte injury was detected in the present study, namely, the production of VEGF-A in cultured podocytes. Podocyte-derived VEGF-A is found to be decreased in sclerotic glomeruli [37], while treatment with exogenous VEGF-A decreases renal sclerotic injuries and restores glomerular capillaries [38]. VEGF-A may serve as a crucial growth factor in maintaining the normal function of podocytes by preventing their apoptosis through the interaction with nephrin and activation of AKT signaling pathway [39]. In the present study, we found that Hcys treatment significantly decreased the production of VEGF-A in podocytes, which was restored by amitriptyline, an ASM inhibitor.

In summary, the present study demonstrated that mice lacking Asm gene produced beneficial effects on glomerular injury and sclerosis occurred in mice lacking Cbs gene with hHcys. This amelioration of glomerular injury by Asm gene knockout or ASM inhibition during hHcys suggests the pivotal role of Asm gene expression and ASM activation in hHcys-induced glomerulosclerosis. These findings may potentially direct towards the development of new therapeutic strategies for treatment and prevention of end-stage renal disease associated with hHcys and hHcys-related pathological processes such as hypertension, diabetes, atherosclerosis, and aging.

## Materials and Methods

### Animals and Genotyping of Mice

Cbs^+/−^ and wild-type mice were purchased from the Jackson Laboratory. We first generated and characterized the mice lacking cystathionine β-synthase (Cbs) and Asm gene by cross breeding Cbs^+/−^ and Asm^+/−^ mice after each of original mouse strains was bred more than 5 generations with careful genotyping to maximize their purity (Figure 1A). Twelve weeks old male uninephrectomized Cbs^+/+^/Asm^+/+^, Cbs^++^/Asm^+/−^, Cbs^+/+^/Asm^−/−^, Cbs^+/−/^Asm^+/+^, Cbs^+/−/^Asm^+/−^, Cbs^+/−/^Asm^−/−^ were used in the present study. In Cbs^−/−^ homozygous mice [40], a genomic fragment of exons 2 and 3, which encodes Cbs putative active site, was replaced by a neomycin selection cassette. PCR confirmation of this genotype was achieved by specific primers designed for wild-type extron 2, 5′-TCTGAGGACCAATGTTAGGATG-3′ and 5′-CTAATGGAACTTCGCCTTGTG-3′. For confirmation of Asm gene deletion [41] in these mice, primers 5′-CTTGGGTGGAGAGGCTATTC-3′ and 5′-AGGTGAGATGACAGGAGATC-3′ were used for genotyping. The genomic DNA was extracted from the mouse tails using the ArchivePure DNA purification kit (5 Prime Inc., Gaithersburg, MD), and the PCR reaction was carried out in a Bio-Rad iCycler, initiated at 94°C for 1 min to denature the template and activate the *Taq* DNA polymerase, followed by 30 cycles of PCR amplification. Each cycle included denaturing at 94°C for 30s, annealing at 55°C for 30s, and extension at 72°C for 1 min. The electrophoresis of PCR products was performed in 2% agarose gel. All protocols were approved by the Institutional Animal Care and Use Committee of the Virginia Commonwealth University.

### High-performance Liquid Chromatography (HPLC) Analysis

Plasma Hcys levels were measured by HPLC method as we described previously [21], [42]. A 100 µL plasma or standard solution mixed with 10 µL of internal standard, thionglycolic acid (2.0 mmol/L) solution, was treated with 10 µL of 10% tri-n-butylphosphine (TBP) solution in dimethylformamide at 4°C for 30 minutes. Then, 80 µL of ice-cold 10% trichloro acetic acid (TCA) in 1 mmol/L EDTA was added and centrifuged to remove proteins in the sample. 100 µL of the supernatant was transferred into the mixture of 20 µL of 1.55 mol/L sodium hydroxide, 250 µL of 0.125 mol/L borate buffer (pH 9.5), and 100 µL of 1.0 mg/mL ABD-F solution. The resulting mixture was incubated at 60°C for 30 minutes to accomplish derivatization of thiols. HPLC was performed with a HP 1100 series instrument (Agilent Technologies, Waldbronn, Germany) equipped with a binary pump, a vacuum degasser, a thermo stated column compartment, and an auto sampler (Agilent Technologies, Waldbronn, Germany). Separation was carried out at an ambient temperature on an analytical column, Supelco LC-18-DB (Supelco; 150×4.6 mm i.d., 5 µm particle size) with a Supelcosil LC-18 guard column (Supelco; 20×4.6 mm i.d., 5 µm particle size). Fluorescence intensities were measured with an excitation wavelength of 385 nm and emission wavelength of 515 nm by a Hewlett-Packard Model 1046A fluorescence detector (Agilent Technologies). The peak area of the chromatographs was quantified with a Hewlett-Packard 3392 integrator (Agilent Technologies). The analytical column was eluted with 0.1 mol/L potassium dihydrogen phosphate buffer (pH 2.1) containing 6% acetonitrile (v/v) as the mobile phase with a flow rate of 2.0 mL/min.

### Morphological Examinations

The fixed kidneys were paraffin-embedded, and sections were prepared and stained with Periodic acid–Schiff stain. Glomerular damage index (GDI) was calculated from 0 to 4 on the basis of the degree of glomerulosclerosis and mesangial matrix expansion as described previously [43]. In general, we counted 50 glomeruli in total in each kidney slide under microscope, when each glomerulus was graded level 0–4 damages. 0 represents no lesion, 1+ represents sclerosis of <25% of the glomerulus, while 2+, 3+, and 4+ represent sclerosis of 25% to 50%, >50% to 75%, and >75% of the glomerulus. A whole kidney average sclerosis index was obtained by averaging scores from counted glomeruli [44]. This observation was conducted by two independent investigators who were blinded to the treatment of experimental animal groups.

### Asm Activity

The activity of Asm was determined as we described previously [13], [21]. Briefly, *N*-methyl-[^14^C]-sphingomyelin was incubated with renal cortical tissue homogenates, and the metabolites of sphingomyelin, [^14^C]-choline phosphate was quantified. An aliquot of homogenates (20 µg) was mixed with 0.02 µCi of *N*-methyl ^14^C-sphingomyelin in 100 µl acidic reaction buffer containing 100 mmol/L sodium acetate, and 0.1% Triton X-100, pH 5.0, and incubated at 37°C for 15 min. The reaction was terminated by adding 1.5 ml chloroform:methanol (2∶1) and 0.2 ml double-distilled water. The samples were then vortexed and centrifuged at 1,000 *g* for 5 min to separate into two phases. A portion of the upper aqueous phase containing ^14^C-choline phosphate was transferred to scintillation vials and counted in a Beckman liquid scintillation counter. The choline phosphate formation rate (nmol·min^–1^·mg protein^–1^) was calculated to represent the enzyme activity.

### Liquid Chromatography–electrospray Ionization Tandem Mass Spectrometry (LC-ESI-MS) for Quantitation of Ceramide

Separation, identification and quantitation of ceramide in plasma were performed by LC/MS. The HPLC equipped with a binary pump, a vacuum degasser, a thermostated column compartment and an autosampler (Waters, Milford, MA, USA). The HPLC separations were performed at 70°C on a RP C18 Nucleosil AB column (5 µm, 70 mm×2 mm i.d.) from Macherey Nagel (Duren, Germany). The mobile phase was a gradient mixture formed as described [45]. The renal lipids were extracted according to previous studies. To avoid any loss of lipids, the whole procedure was performed in siliconized glassware. MS detection was carried out using a Quattro II quadrupole mass spectrometer (Micromass, Altrincham, England) operating under MassLynx 3.5 and configured with a Z-spray electrospray ionization source. Source conditions were same as described previously in our studies and by others [21], [45].

### Cell Culture

Conditionally immortalized mouse podocyte cell line [46], kindly provided by Dr. Klotman PE (Division of Nephrology, Department of Medicine, Mount Sinai School of Medicine, New York, NY, USA), was cultured on collagen I-coated flasks or plates in RPMI 1640 medium supplemented with recombinant mouse interferon–γ at 33°C. After differentiated at 37°C for 10–14 days without interferon–γ, podocytes were used for the proposed experiments as we described previously [21].

### Indirect Immuno-fluorescent Staining

Podocytes were grown on poly-L-lysine–coated chambers and treated with Hcys (40 µM, 24 hrs). In additional group of cells, the Asm inhibitor, amitriptyline (20 µM, Sigma, St. Louis, MO, USA) was added to pretreat the cells for 30 minutes before the addition of Hcys. The cells were fixed in 4% PFA for 15 minutes. After rinsed with phosphate-buffer saline (PBS), cells were incubated with anti-podocin (Sigma, St. Louis, MO, USA, 1: 100), anti-desmin (BD Biosciences, San Jose, CA, 1: 100), or anti-ceramide (Enzo Life Sciences, Plymouth Meeting, PA, 1∶200) antibodies. After washing, the slides were incubated with Alexa 555-labeled secondary antibodies for 1 h at room temperature. After being mounted with DAPI-containing mounting solution, the slides were observed under a fluorescence microscope and photos were taken and analyzed. The fluorescent intensities were quantified by the Image Pro Plus 6.0 software (Media Cybernetics, Bethesda, MD, USA) and the data was normalized to control cells.

### Western Blot Analysis

Western blot analysis was performed as we described previously [36]. In brief, proteins from the mouse renal cortex were extracted using sucrose buffer containing protease inhibitor. After boiled for 5 min at 95°C in a 5× loading buffer, 20 µg of total proteins were subjected to SDS-PAGE, transferred onto a PVDF membrane and blocked. Then, the membrane was probed with primary antibody of anti-desmin (1∶500, BD Biosciences, San Jose, CA, USA) or anti-β-actin (1∶3000, Santa Cruz Biotechnology, Santa Cruz, CA, USA) overnight at 4°C followed by incubation with horseradish peroxidase-labeled IgG (1∶5000). The immuno-reactive bands were detected by chemiluminescence methods and visualized on Kodak Omat X-ray films. Densitometric analysis of the images obtained from X-ray films was performed using the Image J software (NIH, Bethesda, MD, USA).

### Direct Fluorescent Staining of F-actin

To determine the role of ASM inhibition in Hcys-induced cytoskeleton changes, podocytes were cultured in 8-well chambers and treated with Hcys (40 µM, 24 hrs). In additional group of cells, the Asm inhibitor, amitriptyline (20 µM, Sigma, St. Louis, MO, USA), was added to pretreat the cells for 30 minutes before the addition of Hcys or puromycin aminonucleoside (PAN, 100 µg/ml, Sigma, St. Louis, MO, USA) for 24 h. After pretreatment with vehicle, amitriptyline, the cells were treated with L-Hcys (40 µM) for 24 h. After washing with PBS, the cells were fixed in 4% paraformaldehyde for 15 min at room temperature, permeabilized with 0.1% Triton X-100, and blocked with 3% bovine serum albumin. F-actin was stained with rhodamine-phalloidin (Invitrogen, Carlsbad, CA, USA) for 15 min at room temperature. After mounting, the slides were examined by a confocal laser scanning microscope. Cells with distinct F-actin fibers were counted as we described previously [36]. Scoring was obtained from 100 podocytes on each slide in different groups.

### ELISA for Vascular Endothelial Growth Factor A (VEGF-A) in Podocytes

After pretreatment with amitriptyline (20 µM, Sigma, St. Louis, MO, USA), and its vehicle, podocytes were incubated with Hcys (40 µM) for 24 h. A specific podocyte injury compound, puromycin aminonucleoside (PAN, 100 µg/ml) was used to treat cells for 24 h to serve as a positive control. The supernatant was collected for ELISA assay of VEGF-A using a commercially available kit (R&D system, Minneapolis, MN).

### Urinary Total Protein and Albumin Excretion Measurements

The 24-hour urine samples were collected using metabolic cages and subjected to total protein and albumin excretion measurements, respectively [13], [21]. Total protein content in the urine was detected by Bradford method using a UV spectrophotometer. Urine albumin was detected using a commercially available albumin ELISA kit (Bethyl Laboratories, Montgomery, TX).

### Electronic Spin Resonance (ESR) Analysis of O_2_.^−^ Production

For detection of Nox-dependent O_2_.^−^ production, proteins from the renal cortex and cultured podocytes were extracted using sucrose buffer and resuspended with modified Kreb’s–Hepes buffer containing deferoximine (100 mM, Sigma) and diethyldithiocarbamate (5 mM, Sigma). The NADPH oxidase-dependent O_2_.^−^ production was examined by addition of 1 mM NADPH as a substrate in 50 mg protein and incubated for 15 min at 37°C in the presence or absence of SOD (200 U/ml), and then supplied with 1 mM O_2_.^−^ specific spin trapping substance, 1-hydroxy-3-methoxycarbonyl-2,2,5,5-tetramethylpyrrolidine (CMH, Noxygen, Elzach, Germany). The mixture was loaded in glass capillaries and immediately analyzed for O_2_.^−^ production kinetically for 10 min in a Miniscope MS200 electromagnetic spin resonance (ESR) spectrometer (Magnettech Ltd, Berlin, Germany). The ESR settings were as follows: biofield, 3350; field sweep, 60 G; microwave frequency, 9.78 GHz; microwave power, 20 mW; modulation amplitude, 3 G; 4,096 points of resolution; receiver gain, 20 for tissue and 50 for cells. The results were expressed as the fold changes of control.

### Double-immunofluorescent Staining

Double-immunofluorescent staining was performed using frozen slides from mouse kidneys. After fixation, the slides were incubated with rabbit anti-podocin antibody at 1∶100 (Sigma, St. Louis, MO, USA), which was followed by incubation with Alexa 488-labeled goat anti-rabbit secondary antibody. Then, mouse anti-ceramide antibody (Enzo Life Sciences, Plymouth Meeting, PA, 1∶50) was used to incubate with the slides overnight at 4°C. After washing, the slides were incubated with corresponding Alexa 555-labeled secondary antibodies. Finally, the slides were mounted and subjected to examinations using a confocal laser scanning microscope (Fluoview FV1000, Olympus, Japan). All exposure settings were kept constant for each group of kidneys.

### Immunofluorescent Staining

Immunofluorescent staining was performed using frozen slides of mouse kidneys. After fixation with acetone, the slides were incubated with anti-podocin (Sigma, St. Louis, MO, USA, 1∶100), anti-desmin (BD Biosciences, San Jose, CA, 1∶50), anti-nephrin (Abcam, Cambridge, MA, 1∶50), antibodies overnight at 4°C. Then, these slides were washed and incubated with corresponding Texas Red-labeled secondary antibodies. Finally, the slides were washed, mounted and subjected to fluorescent microscopic examination. The images were captured with a spot CCD camera and a pseudocolor was added to corresponding fluorescent image (Diagnostic Instruments Inc., Sterlin Heights, MI, USA). All exposure settings were kept constant for each group of kidneys.

### Statistical Analysis

Data are provided as arithmetic means ± SEM; *n* represents the number of independent experiments. All data were tested for significance using ANOVA for data obtained from multiple animal or experimental groups or paired and unpaired Student’s t-test for two groups of animals or experimental protocols. The glomerular damage index was analysed for statistic significance using a nonparametric Mann-Whitney rank sum test. Only results with p<0.05 were considered statistically significant.
